# GAM4water: An R-based method for extracting wetted areas from remotely-sensed images

**DOI:** 10.1016/j.mex.2024.102955

**Published:** 2024-09-10

**Authors:** Matteo Redana, Lesley T. Lancaster, Chris Gibbins

**Affiliations:** a School of Informatics, Computing and Cyber System, Northern Arizona University, United States; b School of Biological Science, University of Aberdeen, United Kingdom; c School of Environmental and Geographical Sciences, University of Nottingham Malaysia, Malaysia

**Keywords:** Wetted areas, Dry areas, Rivers, Lakes, GAM, GAM4Water

## Abstract

We present ‘GAM4water,’ a R-based method to classify wetted and non-wetted (dry) areas using remotely sensed image indices derived from such images. The GAM4water classification algorithm is built around a Generalized Additive Model (GAM) capable of accounting for non-linear responses. GAM4water can use any type of radiometric data, whether from drones, satellites or other platforms, and can be used with data of different spatial resolutions, geographic extents and spatial reference systems. It is a supervised tool that uses pixel information to distinguish between wetted and dry areas within an image set, extract them and produce a rich output that includes a binary raster, polygons of wetted areas, and a classification performance report. We tested the method in two case-studies, one using high resolution drone images and another using satellite images. The tests show that GAM4water can produce highly accurate classifications of wetted and non-wetted areas, and has the additional benefit of being easily customizable and not requiring complex implementation procedures.

• This paper introduces the first R based method of wetted area extraction for remotely-sensed images.
• The method is based on Generalized Additive Models and is applicable to any remotely-sensed data.

## Graphical abstract

**Figure:**
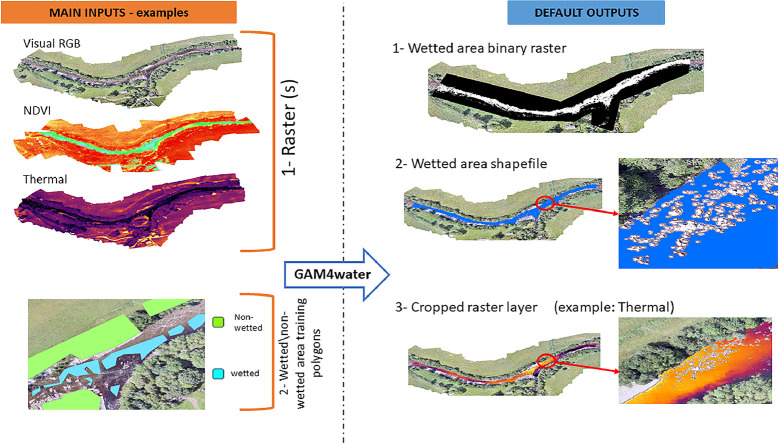

Specifications table

**Table:** 

Subject area:	Earth and Planetary Sciences, Environmental Sciences
More specific subject area:	Remote sensing
Name of your method:	GAM4Water
Name and reference of original method:	NA
Resource availability:	https://github.com/monviso/GAM4water

## Background

The need to distinguish between wetted and dry land is important in many fields of science, notably hydrology and ecology, as well as natural resources management and conservation [1,2]. Satellite images obtained by e.g., Synthetic Aperture Radar (SAR), Multispectral sensors, and Thermal sensors, provide a wide range of radiometric information about the Earth's surface. Such images are commonly used to assess water availability and consumption [3,4], precipitation and aspects of the water cycle [5,6], water quality [[7], [8], [9]] and more recently have been used to explore water temperature patterns [10,11]. The increasing affordability of Unmanned Aerial Vehicles (UAVs) with mouted sensors has created the chance to map and monitor the land surface to an unprecedent level of spatial resolution, potentially providing a greater level of detail about various aspects of standing and flowing water systems [[12], [13], [14]].

One of the first stages in the analysis of radiometric remotely sensed data is to distinguish between wetted and unwetted (dry) areas within an Area of Interest (AOI). Water has a distinct radiometric signature compared to other surfaces; for instance, in the Near Infrared Region (NIR) it shows high absorbance while in the microwave region (typically emitted by SAR sensors) it minimizes the backscatter returned to the sensor. A number of methods have been developed to distinguish between wetted and non-wetted areas, taking advantage of water's distinctive signature [[15], [16], [17], [18], [19]]. However, application of existing methods is constrained by the presence of suspended sediment or algae within the water column, and by shading [16], as well as the lack of required wavelengths in certain types of sensor, especially those attached to UAVs. Advanced machine learning algorithms have been developed to overcome some of these limitations [[14], [20], [21], [22], [23]]. Nonetheless, the implementation of these algorithms outside the context in which they were developed can be limited by the computing skills required. Thus, there remains the need for a relatively simple and customizable tool able to process remotely sensed data automatically and accurately, in order to extract wetted areas within an AOI.

This paper presents GAM4Water, a new tool for wetted area extraction. It uses the R programming language which is widely used within the environmental and ecological sciences and a core pixel classification model based on Generalized Additive Models (GAMs [24]). We provide an overview of GAM4water, as well as details of data inputs, processing, and outputs. We illustrate its use through two case study applications, one to a shallow, wadable temperate river system and another to a large, tropical river. The focus of the case studies is to illustrate the procedures, show GAM4Water outputs and, in particular, present information on accuracy.

## Method details

### Conceptual overview

**Fig. 1:**
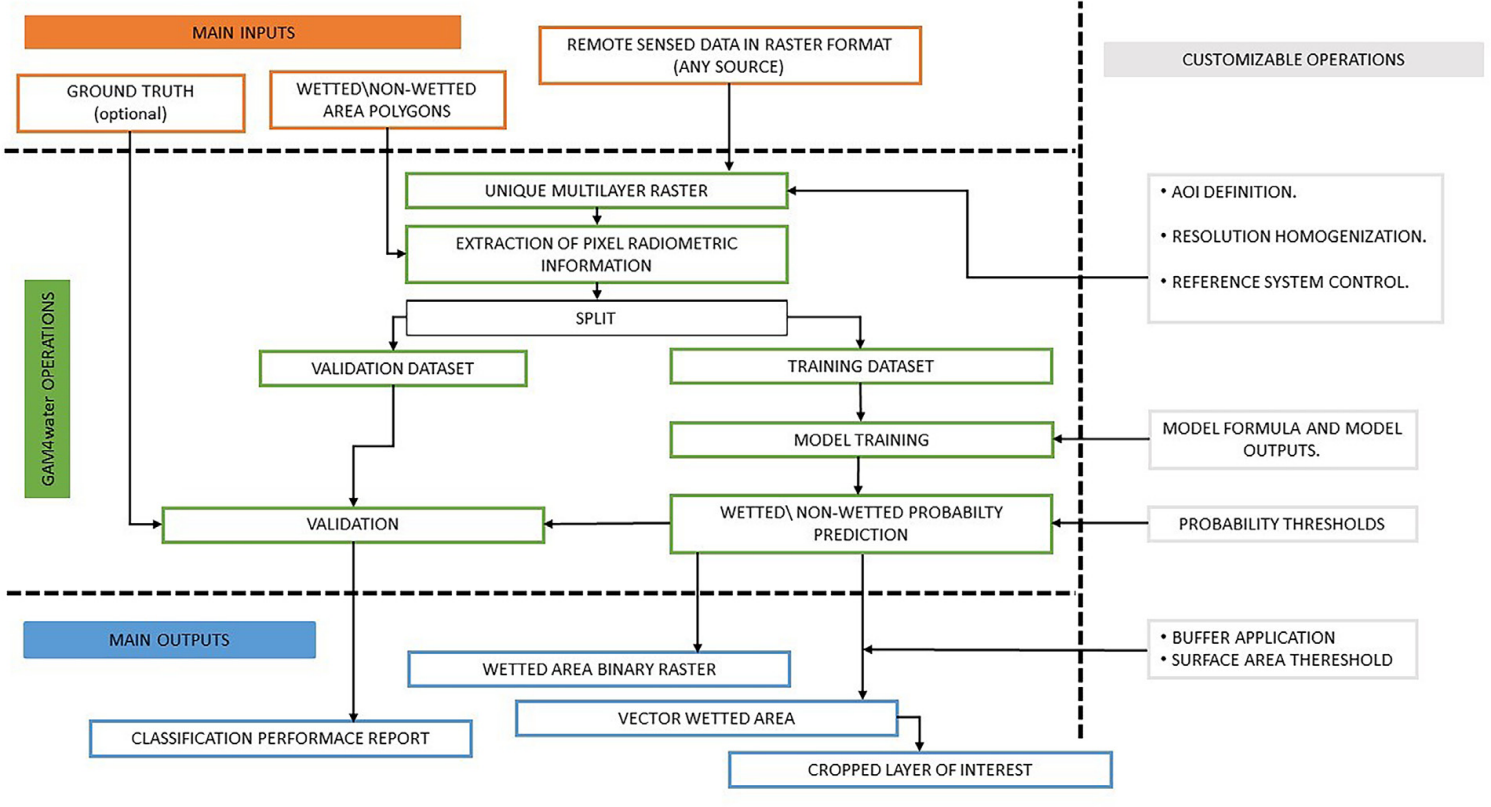
GAM4water overview. The figure shows input data, the main operations performed by the tool, and the main outputs. Aspects that are customisable are indicated.

A schematic overview of GAM4water is presented in Fig. 1. It uses radiometric information from the AOI to perform a binary classification of wetted and dry pixels through application of a GAM; it then automatically produces a set of raster and vector outputs of the wetted area along with classification accuracy metrics. The radiometric information can be any remotely sensed data or derived indeces (e.g., reflectance values at any wavelength, SAR, TIR, grayscale values, RGB /false color images, NDVI) potentially collected from satellite, UAV or aereoplane, and available in a raster (Geo)TIFF format. The classification can be considered semi-supervised, since the GAM training dataset must be provided by the user (see Required data input).

GAM4water uses functions from the R (R [25]) packages terra [26], stars [27], mgcv [24], and sf [28]. A practical tutorial showing the application of GAM4Water is attached to this paper in the Supporting Materials section. A full tutorial is available at the GitHub repository along with the code (https://github.com/monviso/GAM4water.git); this covers all aspects of input data production and GAM4water implementation.

### Required data input

To perform wetted and non-wetted area classification, GAM4water needs 2 main inputs from the user:

(a) One or more single or multilayer rasters containing radiometric information and\or derived indexes (e.g., NDVI, see case study 1) used for classification purposes (e.g., Figs. 4A, 5B). The raster types (e.g., RGB, short\mid\long–wave infrared, SAR,NDVI, SWIR, etc.) and layer number that can be incorporated in the model is only limited by their availability. The case study we present below demonstrate the utility of integrating diverse data input types (i.e., multispectral + SAR from satellite and RGB +NDVI+ TIR from UAV) to improve classification performance. Based on the case studies, we discuss what type of remotely sensed data should be prioritized to obtain an accurate wetted area estimation (see Limitation section). The input raster must have a reference system (i.e., GeoTIFF), that can be different for all of them (see Data preparation, processing and user customizations).
(b) A single (or multi) polygon shapefile, containing polygons of the two categories (wetted and dry areas) within the AOI (Figs. 4B, 5B). The shapefile can be manually produced in any GIS environment and represents the supervised part of the procedure. The shapefile is needed by GAM4water to initialize the classification model. The shapefile must include a column indicating whether each polygon refers to a wetted or dry area.

### Data preparation, processing and user customizations

The following procedures are internal to GAM4water and can be customized by the user (Fig. 1) before launching the function.

GAM4water first homogenizes the resolution of the input rasters, then verifies consistency between their coordinate reference systems and spatial extents. The user can indicate a convenient projection system to transform all the input raster (that, nonetheless, must already have a reference projection system). In case of different resolutions, the user can choose if the raster with lower or higher resolution should be used as reference. This is achieved internally by using the “resample” function of the terra package for R [26]. To speed up computational time, the user can indicate a particular AOI (i.e., a polygon shapefile), which is used to crop the input raster; this is especially convenient for satellite images. To further reduce computation time, the user has the option to indicate an aggregating factor that is used to degrade the resolution of the input raster. This procedure uses the ‘aggregate’ function from the terra package [26] that groups pixels according to an aggregating factor expressed as number of pixels in each direction (horizontally and vertically); the value in the new cell is the mean of the aggregated pixels.

Firstly, GAM4water, stacks all the input rasters producing a single multilayer raster, then extracts radiometric information from all the pixels contained in the input shapefile (i.e., samples of wetted/dry pixels) across each layer of the input rasters. It is worth mentioning here that a larger number of input rasters will increase the computational time, especially for AOIs containing large number of pixels (i.e., large areas or high-resolution rasters). Nonetheless, the amount and diversity of radiometric information can substantially improve the classification performance (see Limitation Section), so there is potentially a trade-off between accuracy and computational time that the user must consider. The radiometric information for each pixel is then stored in a data frame that includes pixel coordinates. At this point each pixel represents a single annotated (i.e., wetted or dry) observation with known radiometric values for each layer/raster.

The data frame is then randomly divided into a training and validation data set (but see Accuracy of wetted area classification), each with a 2/3 ratio of wetted and dry pixels. The training dataset (2/3 of the data) is used to implement a default classification GAM that can have two forms:

(1) $$logit\left(\mu_{i}\right)=f_{1}\left(ly_{1}\right)+...+f_{1}\left(ly_{n}\right)+f_{2}\left(x,y\right)$$

(2) $$logit\left(\mu_{i}\right)=f_{1}\left(ly_{1}\right)+...+f_{2}\left(ly_{n}\right)$$

where $logit(\mu_{i})$ is equal to $\text{log}(\frac{\mu_{i}}{1-\mu_{i}})$, that is the ratio of the probability $\mu_{i}$ of success (i.e., pixel being wetted) to the probability of failure (i.e., pixel being dry) as function of the radiometric values assumed by each pixel at each provided layer/raster (ly). The user can decide if the position (x,y) of a given pixel can be used in its classification as either wetted or dry (i.e., Eq. (1)). This allows the user to account for the often not random distribution of wetted and dry cells, which typically results from the interaction between water volume and bed topography which influences the border between wetted vs dry points across a surface (e.g., lake or river edge). The use of position can be turned on by the user through the initial setting customization within GAM4water. The parameters $f_{1}$ and $f_{2}$, a single smoother and a tensor product respectively, are implemented with a shrinkage version of a thin plate spline function. The shrinkage smoother method [29] shrinks predictor coefficient estimates towards zero. This has been shown to be an effective method of model predictor selection by forcing non-significant effects to 0 and thus effectively dealing with bias caused by predictor correlation and concurvity. The two default models ((1), (2)) are relatively simple, but the user can define more complex equation structure and interaction terms if preferred. The estimation of the smoothing parameters is done using the *fast* Restricted Maximum Likelihood (*f*REML) method [30] through the *bam* option in the *mgcv* package. The function *bam* [[31], [32], [33]] fits a GAM model, but it is designed specifically for large datasets to optimize memory use, and save computational time; it can be run in parallel over multiple processor cores (the number can specified by the user). Optionally, GAM4water can output a summary and a predictor effects plot from the GAM function.

The fitted model (Eqs. (1) or (2)) is then used to estimate each pixel's probability of being wet or dry for the whole AOI, based on their specific radiometric values. In the default set up any pixel with probability ≤0.5 is classified as dry, while those with probabilty >0.5 are considered wet. However, the user can alter these probability thresholds.

### Accuracy of wetted area classification

The accuracy of the wetted area extraction is still computed automatically in the GAM4Water function, following the procedures described in Data preparation, processing and user customizations. GAM4Water allows two methods for assessing the accuracy of the classification of wetted and non-wetted areas. The first is the “default” method, where the classification accuracy is assessed using the validation dataset, that is the dataset formed by the pixels not selected in the training dataset (see Data preparation, processing and user customizations). For this GAM4water establishes which of the pixels in the validation data set were correctly classified in the prediction generated by Eqs. (3)–(5). The second method is the “ground” method; it can be used when a field-based measured area of the wetted area surface (provided to GAM4Water in a polygon shapefile format) is available. In this case GAM4water uses this ground- measured polygon to verify which of the pixels classified as wetted are contained in the “ground” polygon with Eqs. (3)–(5). In both cases the classification accuracy is evaluated based on the binary raster output, thus the following steps (i.e., application of a surface area threshold or a buffer e.g. Fig. 1; see. 2.4) do not affect the accuracy tests.

In both methods, the evaluation of the classification accuracy is based on the F1 score (Eq. (3)), defined as the harmonic mean of precision (P) and recall (R) computed as:

(3) $$F1=2*\frac{P*\;R}{P+R}$$

and

(4) $$P=\frac{T_{p}}{T_{p}+F_{p}}$$

(5) $$R=\frac{T_{p}}{T_{p}+F_{n}}$$

P and R are defined with the concepts of true positives (Tp), true negative (Tn), false positives (Fp) and false negatives (Fn). In the GAM4water context, “positive” is considered a wetted pixel, and “negative” a non-wetted pixel. An F1 value =1 is obtained when a perfect classification is achieved. The metric P quantifies the fraction of truly and classified wetted pixels that are classified as wetted; the metric R quantifies the number of pixels retrieved. The closer P and R values are to 1 the higher the power of the model to correctly identify wetted pixels. The F1 score is then used to indicate model classification accuracy by using the harmonic mean of P and R. The closer F1 is to 1 the more accurate the classification. All information from this accuracy classification is reported in a .csv output file (see Outputs).

The conceptual difference between the two validation methods should be stressed. By using the “ground” method of validation we provide an independent, field-measured, wetted area from which the classification accuracy can be established; this allows a fine accuracy evaluation especially for the pixels in the proximity of the water body's banks (that are known with a precision that is dependent on field measurements). With the “default” method, on the other hand, the accuracy is evaluated on a set of independent pixels within the validation dataset, but these are randomly extracted from the sample polygons provided as input (see Required data input). Thus, there is no control on their position or classification complexity. A hybrid approach could be represented by a manual, accurate digitalization of the “ground-truth” directly on the raster. Nonetheless, as we show in the two case studies, high F1 values resulting from application of the “default” method suggest that it correctly captures the actual accuracy in the extraction of the wetted areas.

### Outputs

**Fig. 2:**
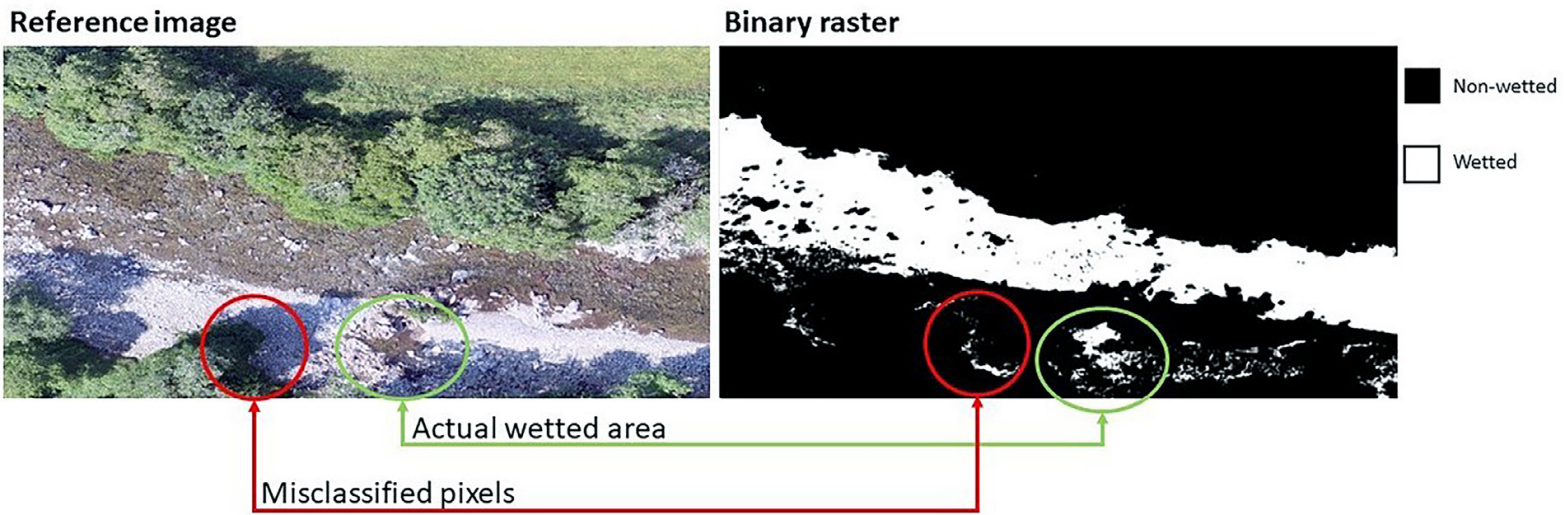
Example of GAM4water wetted area classification applied to a shallow river. In this example, due to shading by trees some non-wetted pixels are erroneously classified as wetted (red circles) due to similarities with real wetted pixels. All adjacent white pixels are vectorized into wetted polygons unless removed by the user using a more stringent probability threshold or a surface area threshold. In this case the area of interest is the main wetted part of the channel, and so the smaller patches (green) of water on the lateral bar can be removed using the area threshold.

GAM4water produces three default outputs that are deposited within a destination folder: (i) a binary raster of the classification where 0 = non-wetted pixels and 1= wetted pixels (note that NA is used to indicate non-classified pixels that have values outside the probability thresholds), (ii) a polygon shapefile of the wetted areas containing a number of polygons corresponding to the number of groups of contiguous pixels classified as wetted (i.e., in the case of standing waterbodies such as ponds and lakes with no islands or emergent areas, or deep rivers, there will be a single polygon; in shallower lakes or rivers with gravel bars, or emergent rocks, multiple polygons will be created – e.g., Fig. 2), and (iii) a .csv file containing a summary of the classification performance (see 2.4).

To fine-tune the outputs to the specific study needs, a number of options can be customized by the user, directly in the GAM4water function. These include: (I) modification of the classification probability thresholds (the default set to >0.5 = wetted areas, see Required data input), to deal with misclassification of cells (e.g., a stricter probability threshold could prevent shaded areas being classified as wetted, Fig. 2); (II) to preserve only water bodies of interest (i.e., remove secondary wetted areas when only the main water body is of interest), the user can set a surface area threshold, below which wetted pixels are not converted into polygons; and (III) specification of a buffer distance that will be used to reduce the wetted area perimeter by a certain number of pixels, to avoid including water body edges in the shape file. The buffer is not a simplification of the output itself, but rather an additional conservative step to remove boundary pixels that can contain a mix of wet and dry areas; this can be useful in situations where the edges of the wetted areas are not representative or contain other sources of unwanted image bias (see study case 1 for an example); in this case both original and buffer-modified wetted area shapefiles will be produced, with the classification performance be based on the original output (i.e., the non-buffer one, see 1.4). Other optional outputs include a cropped version (i.e., solely wetted area) of all the layers used as input.

The spatial outputs are generated using the same reference system set by the user at the optional input stage (see Data preparation, processing and user customizations), or the one automatically detected by GAM4water. Even though GAM4water can work with non-projected coordinate systems, it is advisable to use a projected reference system. Nonetheless, there is the option for the user to export all the desired outputs in a WGS84 (epsg: 4326) reference system.

## Method validation

### Case study 1: drone-based RGB, NIR and TIR images

The rationale for the study was to understand the thermal behavior in the mixing zone between two rivers (the dammed Errochy Water and the undammed Garry River, Scotland), and the implication of this behavior for photosynthetic activity. Thus, visual RGB, TIR and multispectral data were collected around the confluence (56.76523 N, 3.94904 W) on 21/07/2021 at 07:31 and 15:28 hrs. Images were collected using a Zenmuse XT2 radiometric camera (visual RGB + TIR images) coupled with a Micasense RedEdge MX camera (acquiring at 5 bands, from 0.475 µm to 0.842 µm). Both sensors were mounted on a DJI Matrice M210 UAV. The AOI was a short section of the Errochty Water immediately downstream from the confluence, approximately 800 m long; here the channel is approximately 10 m wide and the water shallow, with numerous boulders and cobbles protruding above the water surface. Ground Sampling Distances (GSD) were 3.2, 10.6 and 5.5 cm for RGB images, TIR images and multispectral data respectively. Images were stitched and processed with Agisoft Metashape [34] to obtain visual RGB, TIR and Normalized Difference Vegetation Index, NDVI (computed from RedEdge MX data) orthomosaics of the AOI [35]. In ArcGIS Pro, orthomosaics were controlled and adjusted to ensure consistency in relative and absolute positioning. Part of the study was to determine water temperature distribution patterns, which required separation of wetted and non-wetted parts of the channel.

**Fig. 3:**
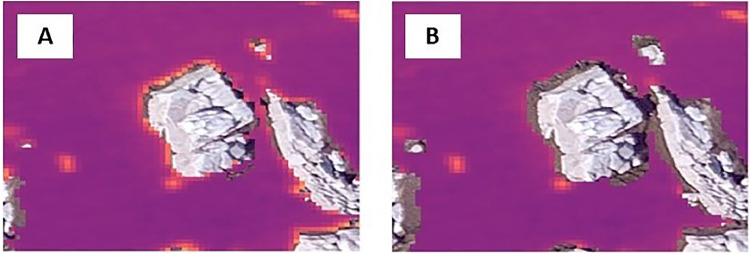
“Halo effect” removal with buffer option. A: Halo effect on TIR images caused by large difference in temperature between rocks and water. B: application of a buffer option (0.2 m) in GAM4water to remove pixel affected by halo.

ArcGIS Pro was used to produce the polygon shapefile to sample wetted and dry areas, one of the two inputs needed by GAM4water (see Required data input). Six rasters were potentially available as radiometric data inputs for GAM4water: the visual RGB orthomosaic (3-layer raster), the two TIR orthomosaics (from images collected at 07:31 and 15:28) and the NDVI raster. The GAM form chosen was Eq. (2) (model without pixel position). We applied a 20 cm buffer to the wetted area, as a non-default option. This choice was because the boundaries of thermally contrasting objects are known to show a “halo effect” (Fig. 3) that does not correspond to real pixel temperature [36] and thus is a potential source of bias when separating wetted and dry areas based on pixel values (Fig. 3).

We applied an aggregating factor equivalent to a surface area threshold of 3000 m^2^ to filter out all small water areas (i.e., puddles) and misclassified pixels. We ran GAM4water under three combinations of the available raster inputs to evaluate their contribution and performance to the classification accuracy: 1- Visual RGB only; 2- visual RGB + Thermal; 3- Visual RGB + TIR + NDVI. The accuracy was evaluated with the “default” method (see Accuracy of wetted area classification). To speed up computation we applied aggregation factors (see Data preparation, processing and user customizations) equal to 4 (GSD = 42.4 CM).

Table 1 reports the classification accuracy for GAM4water applied to AOI. In addition, computational time is reported for each GAM4water input setting run on an ACER Aspire 7 A715–71G-743 K (Intel Core i7–7700HQ 2.8 GHz, 16 GB DDR4 RAM Memory). Note that the computational time (Table 1) includes homogenization of input raster resolution and extent, and reprojection from the original WGS84 to the UTM WGS84 Zone 31 N reference system.

**Table 1:** GAM4water classification accuracy performance for three input scenarios. Comp. time: computational time in minutes and seconds; Pixels: number of pixels used in the validation (“default” method); Tp: true positives detected; Fn: False negatives detected; Fp: false positives detected. P: precision (Eq. (4)); R: recall (Eq. (5)); F1: F1 index (Eq. (3)).

Radiometric information	Comp. time (mm:ss)	Pixel (validation)	Tp	Fn	Fp	P	R	F1
Visual RGB	08′20	502,156	43,135	4728	3600	0.923	0.901	**0.912**
Visual RGB + TIR	14′40	501,857	46,967	858	1179	0.976	0.982	**0.979**
Visual RGB + TIR + NDVI	22′10	563,553	52,880	712	1130	0.979	0.987	**0.982**

**Fig. 4:**
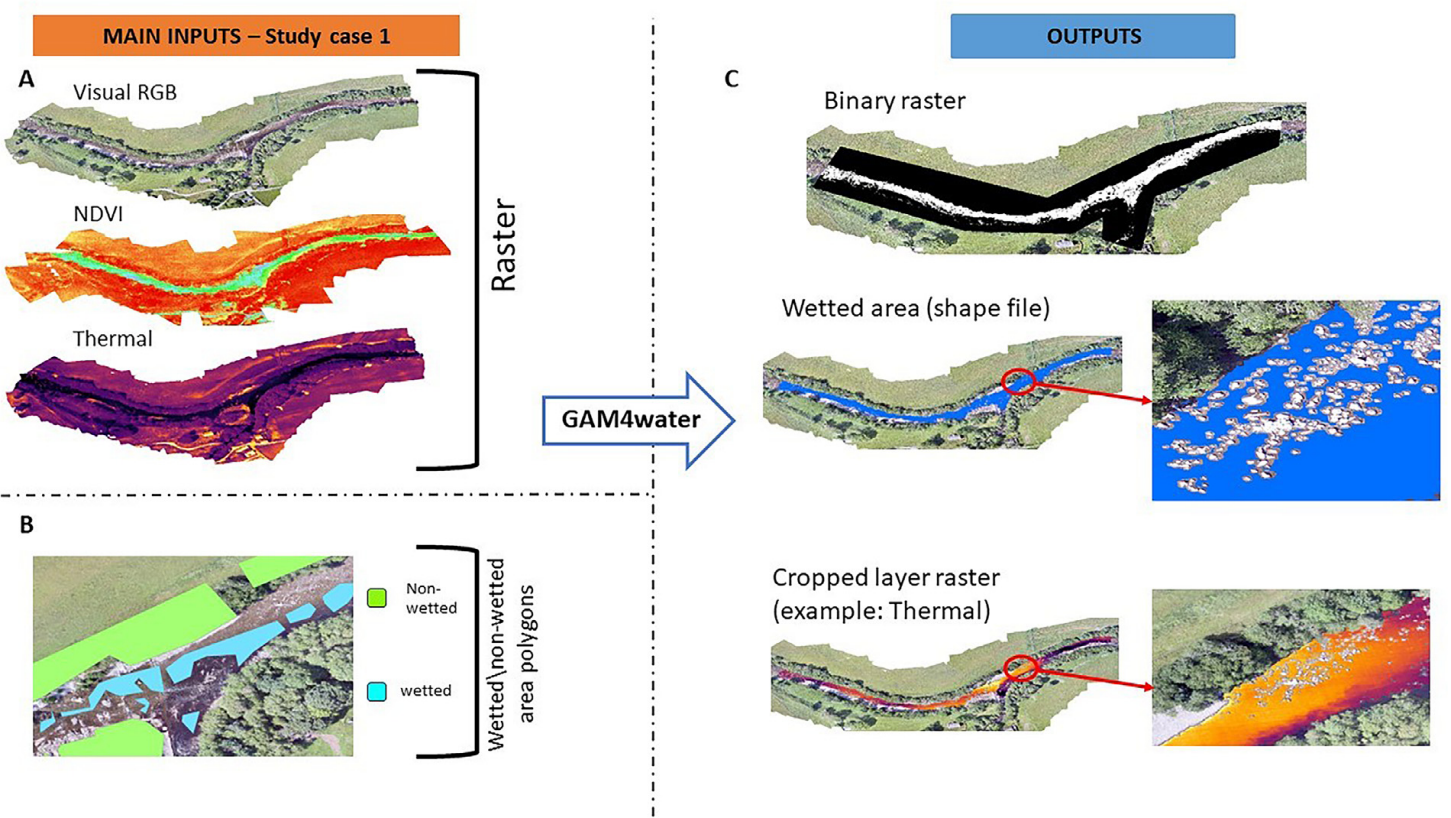
Main inputs and outputs of GAM4water applied to the Errochty Water case study. A: raster inputs; B: subset of the wetted and non-wetted polygon sample shapefile; C: binary raster (default), wetted area shapefile (default), and wetted area masked layers (optional) outputs, shown for the TIR layer.

The combinations of input rasters yielded classification F1 values between 0.912 and 0.982. Classification accuracy increased with the variety of radiometric information provided as input, although the improvement was minimal with the addition of NDVI data. The increase in the computational time of the combined Visual, TIR and NDVI is likely attributable in large part to all the preliminary operations of raster resolution homogenization and spatial extent matching. Fig. 4 shows inputs and outputs from this case study for the classification based on Visual RGB + TIR + NDVI raster.

### Case study 2: Baleh river wetted area from multiple satellite images

The Baleh river is located in Sarawak, Malaysia (1.80505 N, 113.77201E). A large dam is currently being built on the river and is due for completion by 2028. Work to develop Functional Flow (FFs; [37,38]) recommendations to help guide dam operation is underway, and as part of this, data on various aspects of the river have been collected, including a precise estimate of the wetted area boundary needed for FF flow development. We used GAM4Water to extract the Baleh river wetted area, with two data sources tested: Sentinel-2 Band 2 (resolution=10 m, central wavelength=490 nm), Band 3 (resolution=10 m, central wavelength = 560 nm), Band 4 (resolution=10 m, central wavelength=665 nm and Band 8 (resolution=10 m, central wavelength =842 nm) and Sentinel 1 Interferometric Synthetic Aperture Radar (SAR) data at 10 m resolution. Sentinel 1 and 2 data were selected as candidate input rasters as several papers have tested their use for determining wetted areas (e.g., [39]).

The default GAM model (Eq. (1)) was selected and a surface area threshold of 3.5 km^2^ was set to remove any misclassified groups of pixels or minor water bodies from the wetted area polygons. We tested the classification accuracy of the GAM4water separately for each of the three data input scenarios reported in Table 2 using the “default” method (see 2.4), specifically: solely Sentinel 2 Band 2, Band 3, Band 4 and Band 8 (S2-B2,3,4,8); solely Sentinel 1 SAR backscatter intensity cross-polarized band, vertical and horizontal (SAR-VH); combined satellites (S2-B2,3,4,8 + SAR-VH). Satellite input rasters were cropped to a smaller AOI centered on the Baleh River; no aggregation factor was applied due to the relatively low number of pixels already included in the AOI. The same processor as case study 1 was used to run GAM4water. A summary of model performance is provided in Table 2.

**Table 2:** GAM4water classification accuracy performance for three input scenarios. Comp. time: computational time in minutes and seconds; Val. Pixels: pixels used in the validation (default method); Tp: true positives detected; Fn: False negative detected.

Radiometric information	Comp. time (mm:ss)	Val. pixels	Tp	Fn	Fp	P	R	F1
S2-B2,3,4,8	12′50	33,612	13,399	460	395	0.971	0.966	**0.969**
SAR-VH	02′40	33,623	1145	12,716	4462	0.204	0.082	**0.118**
S2-B2,3,4,8 + SAR-VH	15′30	33,515	13,631	228	274	0.980	0.984	**0.982**

**Fig. 5:**
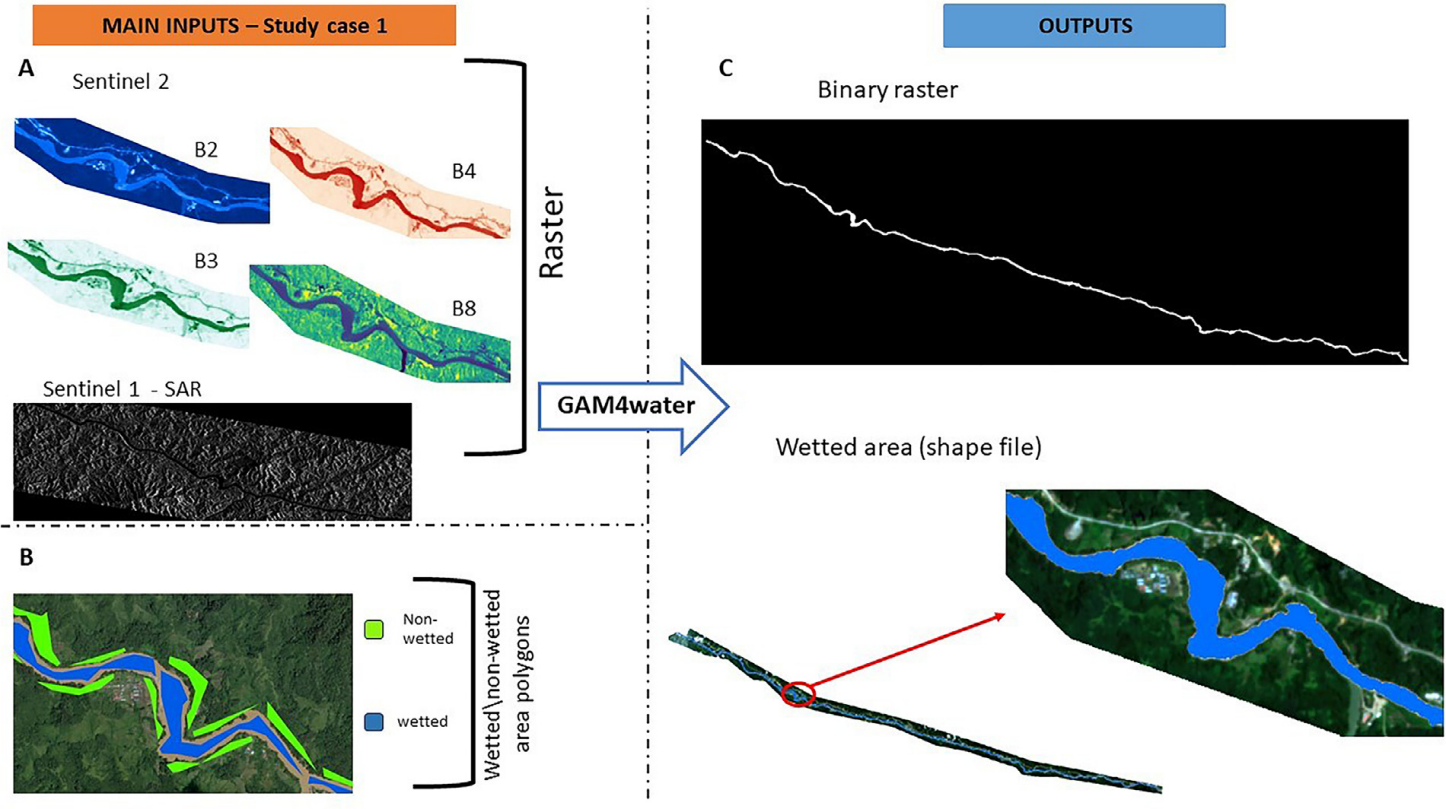
Main inputs and outputs of GAM4water for the Baleh river study case. A: raster inputs (sentinel 2 raster represent a subset of the area investigated); B: subset of the wetted non-wetted polygon sample shapefile; C: outputs required to GAM4water: binary raster (default), wetted area shapefile (default).

The input settings that included Sentinel 2 data produced F1 >0.96, while the model developed using only Sentinel 1 SAR-VH resulted in a poor classification accuracy (F1=0.118). As with case study 1, a longer computation time was found for the model that used different data sources (in this case, S2-B2,3,4,8 + SAR-VH). This is most likely due to the preliminary operations of raster resolution homogenization and extent matching. Fig. 5 shows inputs and outputs from GAM4water for the Baleh River for Visual S2-B2,3,4,8 + SAR-VH data.

## Limitations

GAM4water is an R based tool that is capable of extracting wetted areas from pixel information within remotely sensed images. It uses a Generalized Additive Modelling approach, and includes a series of preliminary steps to ensure consistency in inputs reference systems, resolution and extent, without any action required by the user. A rich default output is produced, that includes a binary raster of the predicted wetted areas, a shapefile of these areas and a summary of the classification accuracy. It offers the possibility for further customization by users. It can run on multiple data sets, and the examples shown here suggest that the best results are obtained when the classification is based on more than one set of layers with diverse radiometric information. Currently GAM4Water accommodate only simple raster; further development could include the integration of tiled rasters.

The two case studies yielded classification of wetted vs non-wetted areas that were generally greater than F=0.9, and up to 0.98. These were based on the “default” method (see Accuracy of wetted area classification) that uses a portion of the pixels given with the wetted/non-wetted sample shapefile to estimate the accuracy. A “ground” based accuracy evaluation would be preferred, considering that it uses the actual wetted area. However, in practice such ground truth data are rarely available; sometimes this is due to the complexity of collecting such data (as in case study 1 where there were multiple emergent stones) or challenges related to the remoteness or physical extent of the area of interest (as in case study 2). Thus, the accuracies reported here may be indicative of those that might be expected in most studies where, out of necessity, the default method is used.

Despite the flexibility to perform the wetted area extraction based on any data input, our case studies suggest that GAM4Water performs well when based solely on Visual RGB data (i.e. in the 0–255 color space). Thus, a useful strategy is to ensure the availability of Visual RGB data for the AOI. Nonetheless, we showed how the integration of the Visual RGB information with other remotely sensed data (such as NIR, TIR and SAR) can substantially increase the model classification accuracy. The accuracy of the classification, as for any supervised classification tool, largely depends on the quality of the training dataset; for this reason, we encourage users to feed training polygons to GAM4Water that extensively cover the AOI, with particular attention to the areas were the classification can be more complex (e.g., shaded areas, high-turbidity water).

The classification accuracy of GAM4Water is comparable with several more advanced and well-established methods of wetted area classification. For instance, the thresholding methods based on the Normalized Difference Water Index (NDWI) can lead to high accuracy, with F1>0.96 [40] but are limited by the need for specific wavelength data (Green and Near Infrared bands, e.g. available in Sentinel 2 and Landsat 8), that are only occasionally available in high-resolution datasets (e.g. from UAVs). The thresholding method applied to SAR data has been shown to be effective but the classification performance is largely context-dependent and the F1 score does not exceed 0.92 [39]. More advanced Machine Learning algorithms are being tested and used for wetted area extraction (and more generally for Land Cover/Land Use classification) from both satellite and high-resolution data (UAV, aerial photogrammetry). These methods, that include Random Forest, decision tree, naïve Bayes classifiers and Neural Networks, can reach classification accuracies with F1>0.96 (e.g., [[20], [41], [42], [43], [44]]). Nonetheless, like GAM4Water, these methods still rely on adequate training datasets; importantly though, their implementation can be demanding in terms of coding needed, and are heavier to run on home-grade computers.

GAM4Water uses an easily accessible coding language, already implemented with a function structure. As well as good classification accuracy, GAM4Water has the capability to deal automatically with inconsistency in the inputs, related for example to their resolution, projection system and extent.

## Ethics statements

No ethical considerations were required.

## CRediT author statement

MR, CG, and LL collected the data presented. MR designed and implemented the GAM4Water function. MR, LL, and CG wrote the manuscript.

## Declaration of competing interest

The authors declare that they have no known competing financial interests or personal relationships that could have appeared to influence the work reported in this paper.

## Appendix Supplementary materials

Image, application 1
