# Supplementary material for: GAM4water: An R-based method for extracting wetted areas from remotely-sensed images
Source: MethodsX. 2024 Sep 10;13:102955. doi: 10.1016/j.mex.2024.102955 (PMC11462176; doi:10.1016/j.mex.2024.102955)
Supplement: Supplementary file 1 [file mmc1.docx]

GAM4Water implementation: practical guide.

- **Step 1**

Download the function code at <https://github.com/monviso/GAM4water>

Open and run the function in R environment.

GAM4Water has been tested under R 4.3.2, Rstudio 2023.09.01 and need packages “terra”, “sf”, “mgcv”, “star”.

- **Step 2: remote sensed data input**

Place in a single folder all the input data (orthomosaics, satellite granules, etc) usable by GAM4Water to operate the classification. GAM4Water can work with any typology of data, single or multilayer potentially with different projection, resolution and extent. Note: must be ensured by the user the correct co-registration of the data inputs.

- **Step 3: input wetted non-wetted sampling polygons.**

An example of wetted/non-wetted polygons digitalized in ArcGIS Pro is reported in the following figure. Yellow polygons cover non-wetted areas and blue polygons wetted area. The polygon can be a gross and quick digitalization of the wetted and non wetted area in AOI. The pixel within each polygons will be then automatically used by GAM4Water to train the classification model and validate the classification (that’s why it’s important to ensure data inputs co-registration)


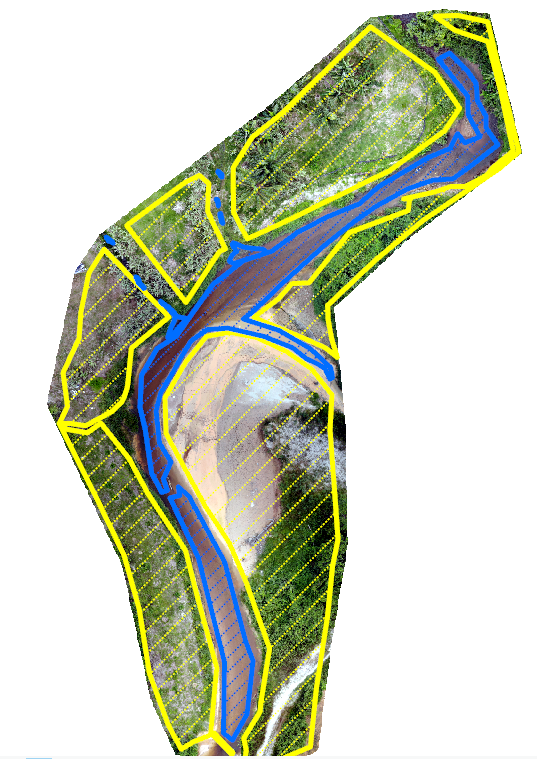


In GIS environment the shapefile should be structured as follow:


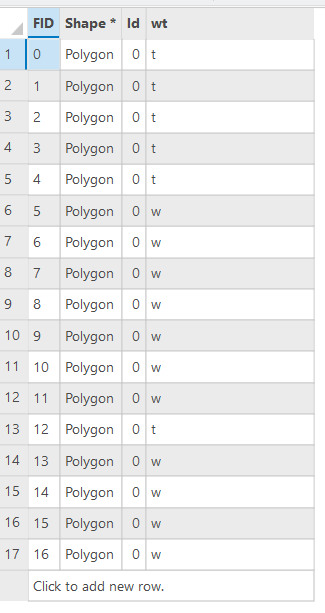


where the column named "wt" indicate if a particular polygon is watered ("w") or not ("t").

- **Run GAM4Water**

Once remote sensed data input and the wetted/non-wetted polygons are ready. The function can be lunched to automatically obtain all the classification outputs. The function consist of all the following arguments (in bold are reported the required arguments).

GAM4Water(**wt_path, Input_paths**, NAval=0, **out_path**, AOI_path=NULL, groud_truth=NULL, respl="low", classID=c("w","t"), ref_crs=NA, xy=T, nth=4, form=NULL, gam_out=F, class_prob=c(0.5,0.5), main_water =NA, buffer=NA, full=F, geogr=F, aggregating_factor=NA)

**Arguments**

| **wt_path** | Character. Complete path to the folder with the wetted/ non-wetted polygons ( e.g., “D:/GIS/polygons/” |
| --- | --- |
| **Input_path** | Character. Complete path to the folder with the remote sensed data input( e.g., “D:/data_input/” |
| NAval | Numeric. Value that must be interpreted as NA in the input data,. Default 0 |
| **out_path** | Character. Complete path to the desired folder where all outputs will be written |
| AOI_path | Character. Complete path to the folder with the option AOI to be used to crop the scene. |
| Ground_truth | If NULL (default), the default accuracy of the classification will be based on the “default” method. If the “ground” method is desired, then a charchter with the path to the wetted area ground truth polygon. |
| respl | “low” (default) or “high”. In case of input with different resolution the lowest (or highest) resolution will be used to uniform resolution |
| classID | Character. Name of the wetted and dry polygons in the input shapefile field. Defluat “w” and “t” |
| Ref_crs | Character. Desired crs of the outputs. Must be specified in the epsg format (e.g., “epsg:4326”) |
| xy | Boolean. If the pixel location must be used in the classification model. If true (default) the model has the form $logit\left( \mu_{i} \right)=f_{1}\left( {ly}_{1} \right)+\ldots+f_{1}\left( {ly}_{n} \right)+f_{2}\left( x,y \right)$, if FALSE the model has the form $logit\left( \mu_{i} \right)=f_{1}\left( {ly}_{1} \right)+\ldots+f_{1}\left( {ly}_{n} \right)$ . |
| nth | Numeric. Number of physical cores available on the laptop/computer for computation. Default 4. |
| form | Character. An alternative formula specified by the user in GAM format. |
| Gam_out | Boolean. If TRUE a summary of the GAM (model performance, predictors relative effect and plots) |
| Class_prob | Numeric. The threshold to use to classify wetted and non-wetted area. By default pixel predicted with a probability >0 and <=0.5 are classified as non-wetted, pixel with probability >0.5 and <=1 are classified as wetted. If thresholds are changed pixel with exceeding probabilities are returned as NA |
| main_water | Numeric. An area value (in meters) below which minor wetted area are discarded. If set, the input raster must be in a projected reference system or one must be specified in “ref_crs”. |
| buffer | Numeric. A buffer value (in meters) to be applied to the wetted area polygons. If set, the input raster must be in a projected reference system or one must be specified in “ref_crs”. |
| full | Boolean. If TRUE, all the input raster are returned cropped with the wetted polygon |
| geogr | If TRUE, all the outputs will be returned in WGS84 format |
| Aggregating _factor | Numeric. If set, the number indicate an aggregation factor for the input raster. Useful for high resolution images to reduce computational time. |
